# Late-life depression in socially and culturally diverse settings

**DOI:** 10.1017/S2045796020000815

**Published:** 2020-09-10

**Authors:** Oye Gureje

**Affiliations:** WHO Collaborating Centre for Research and Training in Mental Health, Neuroscience and Substance Abuse, University of Ibadan, Ibadan, Nigeria

**Keywords:** Depression, geriatric psychiatry, mental health, multicultural

## Global ageing trends

There are striking differences in ageing between populations in the West and those in Sub-Saharan Africa (SSA). Even though population ageing is a global phenomenon with projections indicating that the number of older persons will more than double over the next three decades, reaching over 1.5 billion persons in 2050, these diverse regions of the world will experience vastly different rates of growth (United Nations Department of Economic and Social Affairs, 2020). Thus, while SSA will see a growth rate of more than 218%, the projected growth of the elderly in Europe and North America will be a modest 48%. This differential growth pattern reflects what is already a significantly different profile of ageing in these regions of the world. In 2019, persons aged 65 years and over constituted about 18% of the total population of Western Europe and North America, while they constituted only about 3.0% in SSA. With a life expectancy at birth of about 60 years (compared to over 78 years in Europe and North America), persons who survive to 65 years and beyond in SSA are clearly a highly selected few making their study of potential interest to understanding factors that conduce to survival in otherwise difficult circumstances (United Nations Department of Economic and Social Affairs, 2020). The two editorials (de la Torre-Luque and Ayuso-Mateos, 2020; Ojagbemi *et al*., 2020) in this issue provide an opportunity to reflect on some of such factors as well as on features of ageing that may be universal and those that may be more contextually determined. In this regard, the contrasts between the demographic profiles of populations in SSA and those in Europe and North America are relevant in considering key messages of the studies that the two editorials in this issue have highlighted.

## Depression in diverse settings

The Ibadan Study of Ageing reports one of the highest rates of major depressive disorder in the literature with a 12-month prevalence of about 7% (Gureje *et al*., 2007). This contrasts with an average rate of about 3% reported in high-income countries (HIC) (de la Torre-Luque and Ayuso-Mateos, 2020). The authors note a pattern of higher rates of depression among elderly persons in low- and middle-income countries, compared to those in HIC (Ojagbemi *et al*., 2020). It is interesting that de la Torre-Luque and Ayuso-Mateos (2020) observed that, contrary to the trend for rates of categorical diagnoses of depression to decline with age, the reverse is often the case when a symptom-based approach is used for diagnosis. Whether this is ‘universal and free from culture specific imperatives’ (de la Torre-Luque *et al*., 2020) is open to question as to the literature from some parts of the world, including from SSA, is yet to provide relevant supporting evidence. What seems to be clear from these observations is that elderly persons in LMIC may be at elevated risk for depression.

## Social risk factors

Depression in the elderly is particularly likely to be related to social factors (Vink *et al*., 2008). It is possible that differential exposure to some such social factors may be responsible for differences in the rates of depression between elderly persons in LMIC, especially those in SSA, and those in HIC. In this context, other important demographic megatrends (United Nations Department of Economic and Social Affairs, 2020), including international migration and urbanisation, both of which are probably closely related in the case of SSA, may have different impacts on elderly persons living in these diverse regions of the world. Elderly persons in much of SSA may be experiencing the progressive stripping away of supportive social networks and support with young family members leaving home in search of better economic opportunities.

The Ibadan Study of Ageing suggests elderly persons living in the more impersonal settings of the urban area, who are socially disaffiliated and are lonely have elevated risk for depression. It is likely that the objective condition of social isolation may be the main risk factor with the subjective feeling of loneliness being the intervening feature. The editorial by De la Torre-Luque and Ayuso-Mateos (2020) suggests that loneliness is also a major correlate of depression in elderly populations in HIC. Social isolation and loneliness have been described as a major public challenge among the elderly in HIC (Blazer, 2020). Indeed, loneliness is not only common in communities in the West, affecting between 15–30% of the population (Hawkley and Cacioppo, 2010), but is an increasing global problem which has been described as ‘a hard to detect and lethal behavioral toxin’ (Jeste *et al*., 2020).

However, beyond loneliness, decreasing social network in LMIC, especially in SSA, may have other consequences as well. Specifically, the higher rates of depression in the LIMC may be the cumulative effect of several adverse social factors. Thus, there is the possibility that another important factor could be poverty. Both loneliness and poverty may be linked to reduced informal emotional and material support, the latter being the consequence of a dwindling social network. Elderly persons who lack informal financial support are likely to be at an elevated risk for depression in a setting where formal financial support in the forms of pensions, old age and disability allowances are non-existent. Poverty is very unlikely to be a factor for depression among elderly populations in Western Europe and North America except in some of their more marginalised communities.

Irrespective of how depression is defined, the two editorials suggest that the condition has some similarities mainly in both the western populations as well as in the African population in the ISA. In both groups, there is some evidence that cognitive reserve may be a protective factor. Thus, while a lifetime of unskilled occupation was found to be a risk factor for depression in the ISA, the second editorial suggests that cognitive reserve may be a buffering agent against depression in this age period. The editorials also show that depression is a highly disabling condition among the elderly irrespective of where they live. While the studies reviewed by de la Torre-Luque and Ayuso-Mateos (2020) suggest that disability is not limited to the depressed elderly whose symptoms reach diagnostic thresholds and is equally associated with sub-threshold conditions, the ISA shows that disability persists even after symptomatic recovery. Indeed, in an earlier report of the ISA, it was shown that depression is a more disabling condition than common physical disorders such as diabetes, hypertension and arthritis (Gureje *et al*., 2008).

It is noteworthy that the authors of these editorials have begun to explore the opportunities provided by the unique datasets on the elderly from these diverse cultural and social settings. For example, they have reported that there are broad similarities in psychological and somatic symptom expressions of depression between elderly Nigerians and their Spanish counterparts (de la Torre-Luque *et al*., 2020). There are other potential opportunities and one of such could be to explore the trajectories of depression symptoms in these diverse populations. For example, it would be interesting to determine whether the symptoms of depression also increase with age among adult Africans as reported in this editorial (de la Torre-Luque and Ayuso-Mateos, 2020) and what the implication that might have in regard to the diagnostic construct of the condition in the African elderly population.
